# A study of Iranian immigrants’ experiences of accessing Canadian health care services: a grounded theory

**DOI:** 10.1186/1475-9276-11-55

**Published:** 2012-09-29

**Authors:** Mahdieh Dastjerdi, Karin Olson, Linda Ogilvie

**Affiliations:** 1 York University, Faculty of health, School of Nursing, 4700 Keele Street, Toronto, M3J 1P, Canada; 2 University of Alberta, Faculty of Nursing, 11405 87 Avenue, Edmonton, T6G 1C9, Canada

**Keywords:** Immigrants, Refugees, Health care, Access, Iranians, Canada, Constructivist grounded theory

## Abstract

**Background:**

Immigration is not a new phenomenon but, rather, has deep roots in human history. Documents from every era detail individuals who left their homelands and struggled to reestablish their lives in other countries. The aim of this study was to explore and understand the experience of Iranian immigrants who accessed Canadian health care services. Research with immigrants is useful for learning about strategies that newcomers develop to access health care services.

**Methods:**

The research question guiding this study was, “What are the processes by which Iranian immigrants learn to access health care services in Canada?” To answer the question, a constructivist grounded theory approach was applied. Initially, unstructured interviews were conducted with 17 participants (11 women and six men) who were adults (at least 18 years old) and had immigrated to Canada within the past 15 years. Eight participants took part in a second interview, and four participants took part in a third interview.

**Results:**

Using a constructivist grounded theory approach, “tackling the stumbling blocks of access” emerged as the core category. The basic social process (BSP), becoming self-sufficient, was a transitional process and had five stages: becoming a stranger; feeling helpless; navigating/seeking information; employing strategies; and becoming integrated and self-sufficient. We found that “tackling the stumbling blocks of access” was the main struggle throughout this journey. Some of the immigrants were able to overcome these challenges and became proficient in accessing health care services, but others were unable to make the necessary changes and thus stayed in earlier stages/phases of transition, and sometimes returned to their country of origin.

**Conclusion:**

During the course of this journey a substantive grounded theory was developed that revealed the challenges and issues confronted by this particular group of immigrants. This process explains why some Iranian immigrants are able to access Canadian health care effectively while others cannot. Many elements, including language proficiency, cultural differences, education, previous experiences, financial status, age, knowledge of the host country’s health care services, and insider and outsider resources work synergistically in helping immigrants to access health care services effectively and appropriately.

## Background

Immigration is not a new phenomenon but, rather, has deep roots in human history. Documents from every era detail individuals who left their homelands and struggled to reestablish their lives in other countries. Because immigrants comprise a significant percentage of the population and are less likely to come from Europe than in the past, Canadian society has become more recognizably diverse with respect to ethnic background in recent years. Ng based on Malenfant and his colleague’s report pointed out that in 2006 almost 20% of the population was foreign born and it is expected to reach at least 25% by 2031 [1]. Although immigration from Iran is not widespread compared to other countries, it has grown as a result of the Islamic revolution, American political and economic sanctions, the Iran-Iraq war, and other socioeconomic issues. Thousands of Iranian families have moved to other countries, mostly in North America and Europe, and some have chosen Canada.

Cultural diversity has reciprocal effects on the immigrants themselves and on the host society with respect to culture, including ways of living, values, beliefs, and languages. As a result, the health and the health services needs of this large and growing share of the population are not necessarily the same as those of people born in Canada. Maintaining health and accessing appropriate health services as needed are two challenges faced by all immigrants.

Many studies regarding immigrants’ health have concentrated on psychiatric issues such as posttraumatic stress disorder, depression, mental health, suicide, stress, and adaptation or coping [2-7]. Other researchers have studied acculturation, assimilation, the concept of health, health status, immigration status, poverty, race, gender, structural barriers, lifestyle choices, ethnicity, and lack of cultural competence [8-10]. All of these issues have potential implications related to access to health care resources, but only a few studies have focused on the use of health services by Iranian immigrants/refugees [2-4,11-14]. The study described in this article was the first in a research program focused on learning more about how access to health care resources helps individuals address health-related problems. The research question in this study was, “What are the processes by which Iranian immigrants learn to access health care services in Canada?”

## Methods

### Study approach and procedures

We used a qualitative design influenced by Charmaz’s approach to grounded theory to explore and understand the processes by which Iranian immigrants learn to access health care services in Canada. Grounded theory is based on symbolic interactionism that explains people’s behavior based on their interpretation of specific symbols in their lives [15]. Grounded theory focuses on what is happening with the individual, and between groups of individuals, with respect to a particular context [15]. Using grounded theory helps a researcher understand the meaning of concepts, events, and situations from the perspective of the research participant, to link these elements within a process, and to generate a theory that shows the relationships among these elements [15]. In the constructivist approach, categories, concepts, and the theoretical level of analysis emerge from the interactions between the researcher and the participants [15]. This approach "provides an interpretive portrayal of the studied world, not an exact picture of it" ( [15] p.10). By sharing the participants’ world, the researcher learns more about both the participants’ constructions and his or her own world [15].

### Sample selection and data gathering

Following ethical approval from the appropriate Health Research Ethics Board, two groups of Iranian immigrants were recruited, those who came to Canada independently (not students or through sponsorship) and those who came to Canada as refugees under the terms of the Geneva Convention and were recognized as such by the Canadian government. Additional eligibility criteria included immigration to Canada within the past 15 years, currently living in a mid-size city in western Canada, and 18 years or older at the time of immigration. Participants’ ages ranged from 25 to 49 years and the length of stay in Canada was from 2–15 years with means of 35 years and 7 years respectively (Additional file 1: Demographic Data). Seventeen participants were recruited (11 women and 6 men). Data collection continued until all categories were saturated and no new information was obtained. Data were gathered from February to October 2005.

Because the goal was to understand the process by which Iranian immigrants access the heath care system in Canada, we began with a purposeful sampling approach [16] and interviewed individuals who immigrated to Canada at least two years earlier. As data analysis progressed, additional interviews with more recent immigrants were conducted to strengthen the emerging theory [15-17]. Eight participants took part in second interviews, and four took part in third interviews. Interviews lasted 90 to 180 min. To reduce the potential for misunderstanding, participants were told they could speak either Farsi (Persian) or English. Interviews were conducted, tape-recorded, transcribed, and analyzed in Persian, as all participants preferred speaking Farsi.

The interview started with a very broad opening question: “Tell me about your experiences after coming to Canada.” Interviews became more structured and focused on health-related experiences as the study progressed [16] (Additional file 1: Initial Interview Questions). Because not all members of the research team spoke Farsi (Persian), the first three interviews were translated so that members could gain some familiarity with the data. Back-translation was employed to guarantee the authenticity of the translation. In addition, three participants who were fluent in both Persian and English were asked to read the translated interviews and confirm their accuracy. All three stated that the translations matched what they had said in Farsi (Persian). The translated interviews (English) were coded by one team member (KO), and the Farsi version of these interviews was coded by the first author (MD). As expected, the coding results were similar but not identical (Additional file 1: Example of coding in English and Farsi). Members of the research team discussed the subtle nuances within the codes in the English and Persian transcripts and developed descriptions that expressed concepts from the Persian transcripts in English as fully as possible. In addition, in the second and third interviews, MD shared with participants what she had learned from the first interview to gauge how well she had captured their stories. She also shared concepts that emerged from the data analysis and invited their comments.

### Data analysis

As Morse and her colleague stated, “Without rigor, research is worthless, becomes fiction, and loses its utility” ( [18] p.2). To attain the rigor in this qualitative study, we applied Morse and her colleagues’ recommendations. They mentioned that it is the researchers’ responsibility to establish reliability and validity of a study by applying verification and self-correction. Specifically, we ensured rigor by ensuring appropriateness of the sample, methodological coherence, concurrent data collection and analysis, theoretical thinking, and theory development [18]. The appropriateness of the sample was attained by initially recruiting individuals who were knowledgeable regarding the topic of the study and who had been in Canada for at least two years. Data were gathered and analyzed concurrently so that information obtained early in the study could be used to identify what was known and what we needed to know. The data collected was constantly compared to what was already known so that the interview questions could be narrowed. The results were reviewed in light of existing studies, which helped to show how this study addressed a knowledge gap and developed new theoretical areas.

Data were transcribed using existing Farsi/Persian software (Zarnegar 5.2., a Farsi Word Processor). This strategy facilitated a thorough and systematic approach to the constant comparison required in grounded theory. Coding was carried out on paper copies of the transcriptions, without the use of a computer, because no existing software could accommodate Farsi (Persian). As Charmaz suggested, memos, graphic representations, and diagrams were used to assist with coding [15]. Analysis of the interviews began during each interview and continued through the transcription of each interview, with the preparation of analytical memos related to the formulation of theory [15]. The transcription process itself provided the opportunity to begin coding and preparing memos. The main goal of grounded theory is the generation of a theory, or set of interrelated propositions, that are grounded in the data. Data collection, analysis, and interpretation are simultaneously conceptually driven. In grounded theory, coding (open and selective), theoretical memoing, and constant comparison are fundamental analytic methods. Charmaz considers coding a process of sorting or categorizing data [15]. Data collection and analysis occurred simultaneously.

The first step of analyzing data in grounded theory is coding. Categories are generated at the first level of open coding. Open and initial coding helps the researcher to move from the data to theory [15]. We considered each line of a story or statement told by an interviewee and reduced it to key words and phrases that indicated what was happening in the story. We did this by creating two columns-one showing the story as told and the other showing the coding. The first level of coding forces the researcher to begin to make analytic decisions about the data. In the second level of coding, codes is being collapsed to categories. The second level of coding forces the researcher to identify the codes appearing the most frequently and use them to sort, synthesize, and conceptualize large amounts of data. Coding, then, helps the researcher to develop categories that simultaneously described and dissected the data [15,16].

The second step of analyzing data after coding is memo writing [15]. We employed memos, graphic representations, and diagrams to assist with coding. In theoretical memoing, we recorded ideas we had about codes and the relationships among them, categories, and properties by constantly questioning data and making the connection between what we were discovering in the data and what we knew and had experienced. We applied theoretical sampling to “elaborate and refine the categories in your emerging theory” ( [15] p.96). Theoretical sampling helps grounded theorists gain rich data, fill out theoretical categories, discover variation within theoretical categories, and define gaps within and between categories. Theoretical sampling aims to sharpen concepts and deepen analysis to help the work gain clarity and generality that transcends the immediate topic [15]. We addressed the use of theoretical sampling with respect to sampling adequacy and data saturation earlier. We maintained an interaction between what was known and what we needed to know through the development of a data analysis log. We tested new ideas to see how well they fitted the data collected by constantly checking and rechecking [18].

The last phase in grounded theory is to integrate the analysis. This involves putting the memos and data together in an order that makes sense to the researcher [15]. In this respect, we created the order and made connections for the reader. In this last phase, we attempted to reflect the logic of a participant’s experience in order to provide authenticity. The steps vary, so we sorted the memos by category title, mapping the ways in which the ideas connected and choosing an order that worked for the analysis and the prospective audience. Grounded theory interviews are used to tell a collective story, not an individual tale told in a single interview [15]. The power of the grounded theory methods lies in the researcher’s piecing together a theoretical narrative or model that has explanatory and predictive power [15]. To finalize the model, we sought information in the literature. The emerging model was supported by many studies regarding immigrant and refugee populations throughout the world in general and in Canada in particular [2-14].

## Results

**Figure 1:**
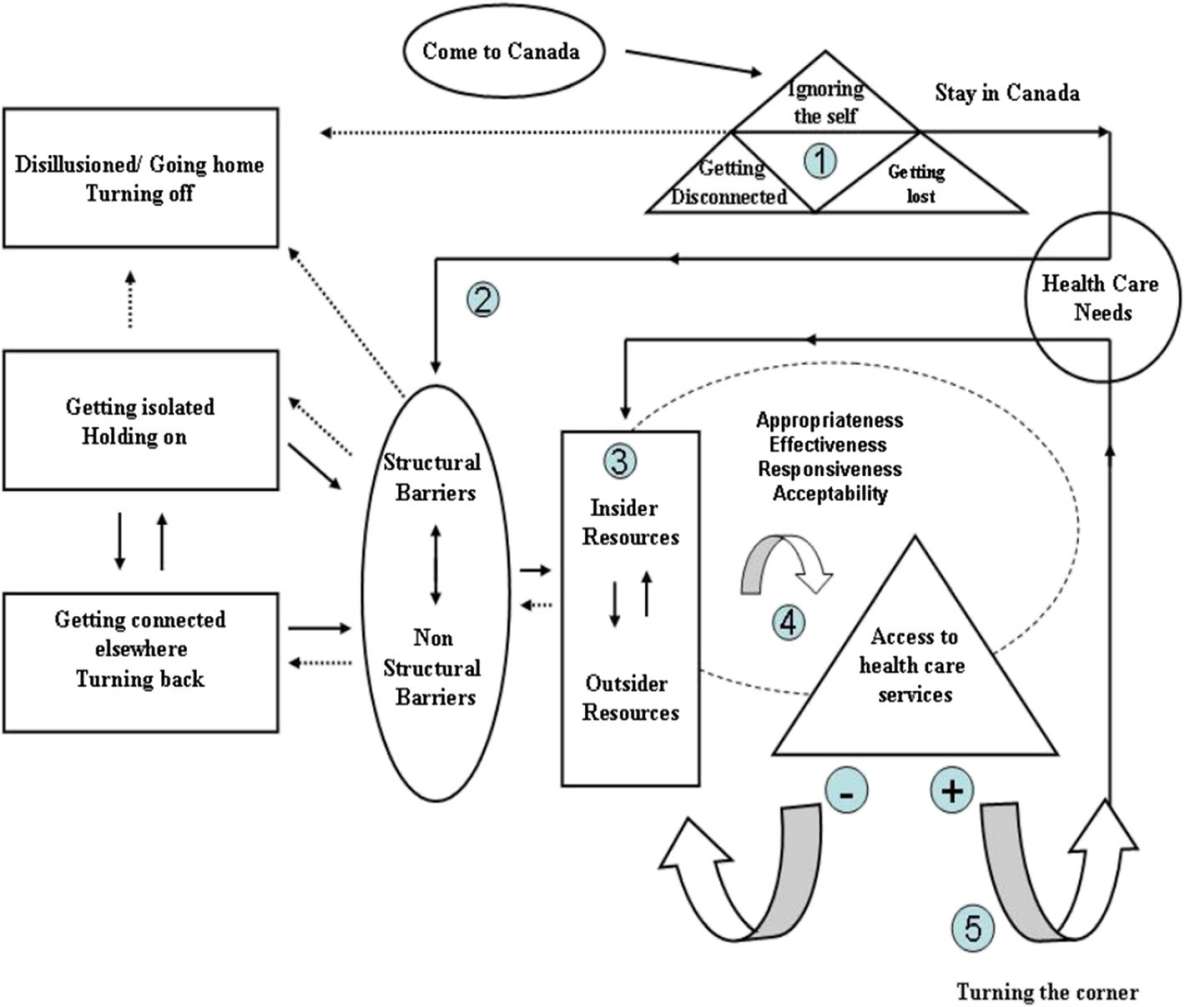
**The Iranian immigrants’ access model to the Canadian health care services.** Note: Language emerged as central to the process of becoming self-sufficient.

Data collection and analysis occurred simultaneously. “Tackling the Stumbling Blocks of Access” emerged as the core category and the basic social process (BSP) was called “Becoming Self-Sufficient”. There are five stages: becoming a stranger, feeling helpless, navigating/seeking information, employing strategies, and becoming integrated and self-sufficient (Figure 1). This process explains why some Iranian immigrants are able to use Canadian health care services effectively whereas others cannot. Each stage is described below, with the support of direct quotes from the interview data translated into English.

### Becoming a stranger: starting point

This stage had three phases: 1) ignoring the self; 2) getting lost; and, 3) becoming disconnected. During this time participants disconnected from the ways they had previously accessed services.

#### Phase 1: ignoring the self

Because of the collectiveness valued in their culture, Iranian participants in this study put the family’s needs first. “Settling in” kept them busy, and they ignored their own health and need for health care.

"I had lots of things to be worried about other than my health: finding a job, learning the language, helping our kids to cope with the new schooling system and their health-there was no extra time to think about myself…I put myself as the last person on the list…I would even say I was not on the list."

#### Phase 2: getting lost

On arriving in Canada, the participants were given many forms and pamphlets about health care in English, French, and other languages that they did not understand. None were available in Farsi. The participants were usually misidentified as being from Pakistan or an Arab country. Information pamphlets were long and usually written in small type. Participants said that they were overwhelmed with information that they did not understand and did not have time to read. Some said that although they could read and understand some English, English was not their first language, and they could not read quickly or understand the information. In addition, for cultural reasons, based on their experience in Iran, they did not really believe the information.

"They gave me lots of paper in small print in 4 or 5 different languages. I guess in English, French, Spanish, Chinese, and, of course, Urdu. I didn’t have any idea about them, and it was Greek to me. I could understand just a bit of English. When I looked at this mixed-up information, I wasn’t eager to read them…even the English version. You know I was so tired and anxious and I got overloaded with a tremendous amount of unnecessary information…It was too much…I got confused…I just put them aside carelessly."

#### Phase 3: becoming disconnected

Most participants mentioned that on arriving in Canada, they felt disconnected from their familiar world. Things did not make sense to them at first.

"Well it was like…I was an ET from another planet. Even making tea and coffee seemed to be different, let alone going to doctor. It took me a long time to stand on my own feet. I didn’t know what I should do or shouldn’t do, or whom to ask. I thought I was living in limbo. I was walking between two cultures and yet couldn’t find my place in either of them … Well, everything was new to me. I felt kind of stupid. I was so nervous. I lost my self-confidence, which made me more incapable."

Many participants knew individuals who found this experience so frustrating that they went back to Iran, leaving their spouse and, in some cases, their children behind. These individuals “turned away” and became “disillusioned”. Those who could not go back to Iran because of their political backgrounds, religions, or financial limitations became isolated.

"My husband just couldn’t take it. He was struggling with every single thing and he didn’t want to change the way he was familiar with. He asked me to return to Iran with my little daughter."

### Feeling helpless: entry point

This stage had two phases: 1) needing access to health care services; and, 2) facing barriers.

#### Phase 1: needing access to health care services

Because immigrants were healthy when they came to Canada and were busy with settling in, most were not prepared to face health problems affecting their children or themselves. Their first contact with the health care system usually arose at a time when their health problems were acute and health care assistance was required quickly. Fear and feelings of hopelessness and helplessness were common as they tried to work out how to deal with Canadian health care services. Limited knowledge of English, financial constraints, and a lack of understanding about how the Canadian health care system worked intensified these feelings.

"[A] day later, around noon, we got up. I felt pain all over my body…I hardly could move. I checked my kids’ temperatures…Oh my god…they were burning like a furnace…I got anxious…I didn’t know what to do…I had lost my self-confidence…I felt helpless."

"It is like a “catch-22” situation with English and health care providers/physicians. It is very overwhelming."

"Well…being in the emergency room because of being in real trouble is different from just dropping by there and getting regular services. Being new and having communication issues make the emergency room like being in hell. When I was in emergency I felt helpless and didn’t know what to do and who to talk to."

#### Phase 2: facing barriers

Each participant faced at least one barrier pertaining to communication/language, finances, cultural differences, and/or time orientation. Language was one of the most prominent barriers to accessing health care services. Immigrants with limited English proficiency could not express what they wanted and verbalize or explain the help they needed to health care providers. In addition, the language barrier barred them from learning how the Canadian health care system worked. They missed doctors’ appointments because they could not communicate with nurses and doctors. Making doctors’ appointments was one of the most frustrating, disappointing, and unsatisfactory experiences.

"I don’t feel comfortable seeing a doctor. They use lots of medical terms that I am not familiar with and I don’t like to ask too much."

"Oh my god going to the pharmacy is a stressful experience. You go there, they talk like a machine gun and talk very fast. I was freaked out."

As a result of communication and language barriers, participants said that they could not trust health care providers and services. In addition, they mentioned that available services did not meet their needs appropriately.

"At the very first when I came to this country, going to the doctor meant nothing to me…I couldn’t tell them what’s wrong with me…I didn’t think that was going to solve any health problems that I had."

"Based on my experience, health care services are running poorly and inappropriately. There is a lack of support from the system, and providers are ignorant or mean. Health care providers need to be more patient and understand their clients and their needs."

"I gave up calling [an interpretation service] because whenever I called them [in the afternoon] to get help and make an appointment for an interpreter, the answering machine started to play a long message. She talked very fast. I did not understand what she was saying and I gave up and did not try it again. This service was not helpful for me. If I could understand English, I would not need an interpreter. I could not understand the reason for having such a long and fast message on their answering machine. I did not try to leave a message because I could not understand it."

Although having an interpreter helped some patients tell their physicians about their problems, they also mentioned the interpretation process as a source of marginalization and discrimination. They felt that physicians almost forgot their presence and communicated with interpreters, not with them. Participants expressed that the physicians should look at and talk to them rather than carrying on direct conversations with interpreters. They stated that it was a humiliating experience and caused them to lose interest in asking for interpreters.

"Although having a translator is very helpful, the problem is that the doctor looks at her when she [physician] asks questions and always talks to her [interpreter]. I am like nobody; I think I am not there. Suddenly, the interpreter is the center of attention, not me. I am the most ignored one and from that point I changed my mind. First of all, I don’t ask for an interpreter; secondly, I try to find people who consider my being as a person and look at me as a whole rather than just language."

Participants stated that some interpreters were not trained and did not have enough knowledge of English. They mentioned that these interpreters could not help them with medical terms and technical words. In addition, they brought out the issue of trust. Participants said that they could not trust an interpreter just because he or she could speak the language.

"Her [the interpreter’s] English wasn’t great and she didn’t have good knowledge of English to tell him [physician] what I really said. She didn’t know medical terminology. My real problem was medical terminology, not simple communication. I could speak broken English …. She [interpreter] didn’t ask my questions. She asked questions that she wanted to know about or she thought were important. I was upset and the doctor got confused too."

There were some situations, however in which patients and interpreters communicated very well and patient advocacy became possible. These interpreters were bridges between patients, health care providers, and health care services.

"Whenever the doctor asked me something and I nodded “No” she thought that was a “Yes,” and Mrs. L always corrected it (because she was Iranian too). The doctor was confused in my “yes” and “no” response. For showing yes and no, we shake our head up and down but people here, for yes they shake their head up and down and for no just move their heads horizontally ___ like this ___ (laughing)."

Individuals who did not have problems with language faced different issues. This group mentioned that they had exposure to health care services other than the Iranian system while they were in European and North American countries, but they still struggled to learn about the new health care system; it took time, but finally they made it through. Although they did not mention feeling hopeless and helpless at this stage of the process, they felt overwhelmed, exhausted, and sometimes even burned out. They mentioned that trying to get information from different sources was a chore. They had to find out what to do and how to do it. This was not an easy job, and it took a lot of energy and time.

"Well, it wasn’t my first experience using different health care services. My first experience was while I was in Germany with my family. They have a different system as well ___different from here [Canada] and from Iran. To tell you the truth over there [Germany], although I had language and cultural barriers and financial limitations, the system was much easier to go along with___ Well we got lots of help and support from friends and government; without those I couldn’t have made it at all. Even with all that help because of having a different language and culture there were moments that I felt so hopeless and frustrated. When coming to Canada the first thing I knew was___ oh yeah ___ everything was going to be different. It was a very overwhelming, and time-and energy-consuming chore. One thing helped me a lot to go through it better than my first experience in Germany was applying some of those strategies such as learning the language, communicating with people, asking them what to do, or telling them what you need."

Participants noted that if hospital staff and other health care service personnel were not sensitive to the beliefs and values of non-white English-speaking immigrants, immigrants would avoid using the services and thus put their health at increased risk. The lack of awareness of cultural differences caused dissatisfaction and a sense that one was not understood, and was being ignored and/or discriminated against.

"Some questions and behaviors showed me that they [health care providers] don’t understand and have no knowledge of other cultures ___ it got on my nerves. Some of them, especially the young ones, thought that if something was different it was odd."

From the standpoint of Iranian culture, one does not ask for help. Rather, one waits to be provided with information. Many participants stated that because information regarding access to health care was not provided, they did not know what to do when they became ill. They did not talk about their needs until they were asked if they needed help. In addition, they thought that if they asked for help, they would not be considered capable, and so by asking they would lose “honour” and “face”.

"We don’t ask…we are waiting to be asked… .to be polite, we always were told to wait until you are asked or try to find a way by yourself."

### Navigating (transition point)

This stage consisted of three phases: 1) realizing the need to know; 2) discovering the differences in the health care system; and, 3) searching for resources.

#### Phase 1: realizing the need to know

After facing health problems or being in need, participants started looking for information and asking for help. Individuals who had access to the Internet and were proficient in English could seek information through reliable sources and were reliable resources for immigrants who did not know the language and did not know how the Canadian health care system worked.

"Well, I didn’t have any problem understanding the language, and I was capable of using the computer. I went to the Health Canada web page and tried to find what to do and where to start. I got the list of doctors, their contact numbers and their addresses. Then, I called and tried to find the best match. Gee, I like it in here. You can get all the information you need in a second. They are very organized."

#### Phase 2: recognizing the differences in the health care system

Participants became aware that in Canada the family physician was the main point of entry into the health care system and that if they wanted referrals to specialists, they had to obtain this referral from the family physician. Since individuals have a greater control over the choice of physicians in Iran, the referral system in Canada triggered feelings of losing the power over decisions concerning their bodies. They found that they could not negotiate as they did in Iran. In Iran people choose their physicians based on whom they trust, but in Canada they did not know anything about their physicians. Participants stated that they found it difficult to trust physicians whom they did not know. Thus, some ignored their health problems or tried to use medication that they had brought from Iran. They did not use herbal medications available in Canada because they were expensive and, again, they could not trust things that they did not know anything about.

"I don’t like it here. It seems your family physician has the right to make all decisions on your behalf…yeah…they discuss the situation with you but they don’t give you lots of choices…there is no room for negotiation…You are caught between a rock and a hard place…no room to make any different move…  .  . You have to go with your family physician’s suggestions."

"I told my doctor that I wanted to choose my specialist but she didn’t listen to me. Well, I didn’t go there. In fact, I changed my family physician. Well there was no point having her. She didn’t listen to me; she had always something more important to think about. I went to a walk-in clinic and from there they sent me to another specialist."

#### Phase 3: searching for resources

After they had learned about the differences between health care services in Canada and in Iran, participants started searching for resources. Participants who had language barriers relied on families, relatives, friends, and people who could speak Farsi (Persian). These “insider” resources sometimes gave wrong or incorrect information and created more problems, or were not helpful. As a result, these participants were dissatisfied and became upset. Because of language barriers and a lack of knowledge about health care services, they kept switching from one person to another to get information. The participants liked to have health care providers from the same country or who spoke the same language as they did. They were looking for Iranian physicians and health care providers and felt more comfortable getting help from them.

"The first thing that struck my mind was calling a friend and asking him what to do. Well it wasn’t a great help. He said that___ well, I don’t know…It didn’t happened to me…"

Although some participants had language barriers, they chose to connect with Canadians (outsiders) to get access to health care services.

"She [the landlady] asked me “have you picked a family doctor yet?” I said “no.” She said “you had better pick one.” I told her “I don’t know how…I don’t know any doctors in Canada…how can I choose when I don’t have any idea … how can I trust them since I don’t know them”… She said, “I like my family doctor, she is very caring. Would you like me to call and see if she accepts new patients? We can go and visit her. If you don’t like her, we will try another one. Don’t worry I’ll do my best to hook you up with a good doctor."

Some participants mentioned that they used insiders (Iranians), some used outsiders, and some chose both outsiders and insiders. They felt satisfied if they got good support and help but became upset and even ignored their problems when they did not get enough assistance. Those participants who did not have language and communication barriers usually used outside resources and were satisfied with the services provided.

"I get help through friends and librarians. To tell you the truth librarians are doing a fantastic job and nobody seems to be familiar with their job. I always tell my friends if you have questions “Go to the library and ask them…they are such wonderful sources."

### Employing strategies and the journey of going through: turning point

This stage consisted of two phases. Phase 1 included weighing options and dealing with barriers, whereas phase 2 related to evaluating outcomes.

#### Phase 1: weighing options and dealing with barriers (turning the key)

After recognizing the differences in health care service provision in Canada, and facing problems and barriers, participants moved to the next step of weighing options and dealing with barriers. Individuals who did not have previous experience with health care systems in other countries and who had language barriers found this phase much more difficult than those who had previous experience or who experienced no language barrier. Some individuals found it so hard that they turned back to the previous stage (Navigating) and could not progress. This group of people could handle some of their health issues but were never satisfied and had to ignore some of their problems. Some even turned away completely, disconnected themselves from Canadian health care services, and reconnected to the health care services in Iran.

"I never could make it. There is always something that stops you. There are lots of barriers. You solve the first, you face the next…Well, I tried to get help from friends and always go back to them and use different ways, but always it seems there is something that makes you feel handicapped. You never win. It is kind of going back and forth. It is frustrating but you get used to it."

Sometimes, participants found the services they required in other countries, often by trial and error. They tried to get help from Iran or asked for help from their family and friends in other countries.

"I brought all the medication I needed for at least 2 years with me (from Iran). I knew I would have no money to buy any medication. Before coming to Canada I heard that medication is very expensive in Canada and it is impossible to afford it."

"Every year, my family sends me some pills for headache, muscle ache, fever, cold and diarrhea and also some routine antibiotics. Therefore, I can manage some common health problems without being in lots of stress to make an appointment to see a doctor and stay in a long waiting list."

Some individuals without exposure to health care services in other countries happened to meet people here who were comfortable with the Canadian health care system. These individuals served as guides or role models, and helped the new Iranian immigrants obtain the services they required.

"This time, I mean my second experience at the doctor’s clinic was totally different from my first experience. Having Mrs. L beside me who knew me and was familiar with my situation was such a big relief, put me at ease and gave me lots of self-confidence. At this time, I could answer almost all of her questions. She was so patient and she spent a lot of time with me."

A third group of participants without exposure to health care services in other countries did not want to learn English and tried to find Iranian doctors. They wanted to speak Persian in Canada and relate to their Canadian physician in a manner that was consistent with the patient-physician relationship in Iran. Having an Iranian doctor was not always a satisfactory experience, however. One participant who had been a physician in Iran and who was currently working as a physician in Canada felt that some Iranian patients had unrealistic demands and expectations of Iranian doctors just because they were Iranians.

"Sometimes they ask us [health care professionals] to do something that is not acceptable and does not fit with Canadian health care services. For instance, they do not have insurance coverage but they don’t want to pay for their visit. They ask me to treat them for free and not report it."

Some participants mentioned that because of less paper work and similar waiting time, they preferred to go to hospital emergency departments, even if their problems were not urgent. They said that it was very convenient for them to drop by the emergency department any time, especially in the evening or late at night, because they did not have to take leave from their job. As well, this meant that they were not under pressure to find someone to take care of their children, as their wives/husbands or their neighbors were available to give them a hand. In addition, they could get their medication free from the department.

#### Phase 2: evaluating outcomes

Participants who could not master English became stuck in this phase; these individuals were dissatisfied, felt marginalized, and perceived discrimination. They did not trust Canadian health care services. Participants perceived being discriminated against and being marginalized while being treated by physicians. They mentioned that misunderstanding, mis-communication, lack of cultural knowledge, and physicians’ self-centeredness could be reasons for discrimination and marginalization. Therefore, they put off visiting physicians, did not follow their recommendations, or got help from informal resources. By doing so, some ended up in critical situations. Some individuals mentioned that mismanagement and provision of inappropriate resources resulted in patient dissatisfaction and perceptions of discrimination.

"The stronger accent you have and the darker you are, the more discrimination you will experience in obtaining health care services."

"I don’t like it here [Canada]. As soon as you open your mouth and speak with an accent, they start asking where are you from and right away about political turmoil in my country…the center of conversation always changes from my problems to my previous country[Iran]…It is really annoying…"

However, many participants stated that time helped them recognize their issues, manage the system, and overcome any shortcomings.

"Little by little…gradually, by facing some problems, we learn that we should take this information into account seriously, and we come to understand that some papers are really important and should be read, and if it says for more information call or contact us at this number without any hesitation…they mean it. The rest is up to us…I mean it is our responsibility to read and follow the directions…it is our job to ask if there are any questions or if we need more information…nobody can read between the lines…they cannot read our minds…if we don’t ask…"

"At first I was very hesitant, but as time went by I learned that if I don’t ask, nobody will know what I need, and if you ask for help or some information, it doesn’t mean that you are not able to take care of yourself. I learned to talk about my concerns."

Over time, participants learned that if they wanted to obtain some health care services, they would have to find a way to trust physicians who were accepting new patients. Because their familiar strategies for developing trust in a physician were not possible in Canada, they developed new strategies. These strategies included looking for physicians who could communicate well and who were willing to spend more time with patients who had special needs related to language barriers, cultural differences, and financial limitations.

"Our doctor in the medi center was a good doctor. We always tried to find out the dates he was working and go for treatments then. He is a really good doctor. He spends lots of time with his clients. He is not just doing his job like a machine. He communicates with his clients very well. You know-you could count on him."

"Well, I came to Canada about 10 years ago, the situation was different…doctors were more approachable and they spent lots of time with patients especially with those who had language barriers."

In this way, Iranian immigrants became able to trust their health care providers in Canada. This trust was built and maintained through experience and over time. Participants who found physicians whom they could trust noted that they were now learning how to manage their new health care problems.

### Becoming integrated and self-sufficient: turning point

Those who tried to accept differences in provision of health care services in Canada and tried to be active contributors in their life felt comfortable accessing health care services. Nevertheless, they mentioned that it did not mean that Canadian health care services do not suffer from any shortcomings. They stated that they were dealing with the services in the same way as Canadians, who have the right to stand up for their rights and get services. Generally speaking, they mentioned that they were pleased with Canadian health care services. They believed that family members or friends who were knowledgeable were great sources of help for linguistically and culturally diverse individuals. These participants stated that they know how to “turn the corner” and deal with new situations. They mentioned that they are ready to help newcomers/immigrants, show them how to deal with life in their new country, and help them to gain independence and self-mastery by empowering them rather than helping them all the time and keeping them reliant on others for resources. They pointed out that newcomers need both inside and outside resources and support. In addition, newcomers should be connected to individuals from their communities who are integrated into their host country and could serve as role models.

"I appreciate Canadian health care services. I’ve gotten good care, and I am more than happy to help people who are in need and to use all available services."

Those who were dissatisfied with services stated that overall they do not like the Canadian health care system and never felt comfortable using it. They said that there is no Iranian community as such from which they can ask for help or get real support.

"I have received health care services for at least 5 years. I am not really satisfied with the health care services, because some providers were not nice to me."

These individuals were skeptical and unable to trust the services. As time passed, this group of people turned away and did not receive services. They left the country or reconnected to the home country’s health care services, or else they turned back and received help from friends in Canada, outside of Canada, and in Iran. In the meantime, if they happened to meet people who are comfortable using Canadian health care services, they tried to follow their suggestions, trusting them and accepting them as role models. Therefore, after a while, some became comfortable using Canadian health care services and took on the role and responsibility of helping others.

"My attitude toward health care services here [in Canada] is different from the time I arrived in this country. Like day and night…Now, I use health care services like a citizen. I don’t have that much problem. I know where to go and if I don’t understand I ask them to explain it. I don’t ignore it as I did before. I don’t hide my feelings and I speak out. One of my most helpful strategies is to obtain some information-as much as I can-before I actually ask any questions or explain my situation."

These findings showed that in the journey of learning to access Canadian health care services, Iranian immigrants went through a process that began with being a stranger and ended with becoming self-sufficient. In this transition, they faced many barriers that they had to overcome. Because of the challenges, some participants found Canadian health care services unsatisfying and untrustworthy. Therefore, they delayed treatment and failed to use, misused, or overused resources and services. Tackling the stumbling blocks to access was the main struggle in this journey. Some of them won on this battlefront and became self-sufficient, but some could not make it and stayed in different stages/phases, going back and forth, living in limbo, dissatisfied, and sometimes even becoming disillusioned.

## Discussion

One of the challenging barriers that immigrants need to overcome is access to health care services. In this study, the core category of “Tackling the Stumbling Blocks of Access” and the basic social process (BSP) of “Becoming Self-sufficient and Integrated” show how Iranian immigrants learned to address this problem. Analysis from this study shows that almost all Iranians in the first stage of transition feel like strangers who are disconnected from the new world. They have little sense of themselves in relation to the new country. They feel incapable of managing their lives, which affects their sense of identity. More importantly, they cannot use their own language in their everyday lives. Therefore, both physically and symbolically, much that they use to construct their world needs to change [3,13].

In accessing health care services in Canada, Iranian immigrants face many challenges and barriers, both structural and nonstructural (communication barriers, cultural differences, financial limitation). In some cases, these barriers are so frustrating that they decide to return to their own country (disillusioned). Of those who stay, some feel helpless and frustrated, and isolated (holding on). These people remain in Canada but reconnect to their country of origin through families, relatives, and friends, and try to meet their health needs by obtaining medications and advice from Iran (turning back).

The potential for isolation among immigrants has important health implications. The feelings of strangeness and lack of belonging contribute to a greater level of disconnectedness from community [19]. Individuals understand each other only through interconnectedness with one another regardless of race, color, religion, or ethnicity. Learning happens in a relationship with others, not in isolation [20]. Disconnectedness from history, belonging, and culture and lack of interconnectedness can lead to physical and mental health problems. The results in this study are similar to those of Jafari and Emami, who reported that the loss of supports, networks, possessions, and meaningful attachments compromised immigrants’ physical and mental health [4,13]. In the study described in this paper, we found that as participants became more isolated they ignored their health problems until these problems became critical (holding on). Although this group of people physically lived in Canada, they obtained most of their medical needs from Iran, which further complicated their care and compromised their health, partly because there was no reliable ongoing record of their health issues. Other authors have reported that immigrants are as healthy as their Canadian counterparts at the time of arrival but that their health status declines, possibly due to stress and unhealthy lifestyles [21]. The findings of our study extend the possible explanations for the “healthy immigrant effect” whereby new immigrants tend to be healthier than their age cohort on arrival in a host country but experience health declines as settlement proceeds. Declines in the health status of our participants appeared to be related to their decisions to ignore their own health issues because of other pressing concerns, often related to settlement priorities, and to the difficulties associated with accessing the Canadian health care system as health problems emerged.

With regard to seeking information, Iranians adopt different methods based on their education, ability in English, and previous experiences. Generally speaking, Iranians are family-centered and prefer to get help from their families rather than from others [12,13]. Those who have communication barriers try to get most of their information from families, relatives, friends, or other Iranians living in Canada. Some, although they have communication barriers, prefer to get help from public services and Canadians. In seeking information, Iranians also try to get help from other resources, such as pamphlets, Iranian satellite broadcastings, Internet, and librarians. In fact, almost all Iranians, with or without communication barriers, rely on librarians as one of the most reliable and helpful sources of information. These findings have not been reported in other studies. Because of this access to information and resources, they feel empowered.

The participants in this study sought the assistance of individuals inside and outside their community to access the health care system. They often found the process of going between “inside” and “outside” frustrating, involving a lot of trial and error. Although interpreters are one of the available resources, the participants in this study had some concerns about the quality of interpreter services. Other authors have noted that although immigrants who have language barriers ask family members, children, or friends to be their interpreters, they often do not feel comfortable with this option [22], as it jeopardizes privacy and confidentiality. Some participants in this study mentioned that they were afraid or ashamed of revealing signs or symptoms of their health problems in front of a family member or friend, especially in front of the opposite gender.

Lack of trust and fear of disclosure are elements that could affect client-provider relations [23]. Clients may try to keep some of their issues private and intentionally misrepresent their symptoms to save face in front of people in general and their community in particular. Since they do not want to talk about private matters in front of friends, they may not answer some questions or may give the wrong answer. Although trusted family members and friends can be used as interpreters to help patients obtain services, these individuals may have limited English proficiency, and thus may misunderstand or misinterpret symptoms, particularly when the terms used are linguistically and culturally bound. For these reasons, mere translation or interpretation by untrained individuals only might not be helpful and could actually make things worse. Additionally, although professional interpretation may help to ease communication between health care professionals and patients, it prevents patients from being active in the dialogue. Our findings are consistent with others who have reported that interpreters sometimes act as information gatekeepers, make decisions, and select what kind of information should be exchanged, and that they often bring their own beliefs and their personal agendas into the interaction [24,25].

The participants in this study wanted to be independent, and so they put considerable effort, energy, and time into educating themselves, learning English, and finding out how the health care system worked. These findings concerning the different strategies used by Iranian immigrants in navigating Canadian health care services are supported by Leduc and Proulx, who studied the pattern of health services utilization by recent immigrants from Algeria, the Philippines, and Sri Lanka living in Montréal, Canada [26]. They found a similar triphasic pattern of health care service utilization, reporting that families relied on a variety of resources and information in each phase of adjustment. Interestingly, although participants were from different countries, the utilization patterns were similar.

Although the majority of health care providers do not intend to discriminate, even “well-meaning people who are not overtly biased or prejudiced typically demonstrate unconscious negative racial attitudes and stereotypes” ( [27] p.15). In this study, participants reported a sense of inferiority and perceived discrimination related to having an accent, dark skin, and being Iranian. As a result, they lost trust in the Canadian health care system and were reluctant to seek health care services. This finding is similar to those of others who have reported relationships among perceived discrimination, negative attitudes, trust and delays in access to care by ethnic minorities [24,28,29]. Fear of being deported may also influence immigrants’ trust of health care providers. The issue of trust is important because the mistrust of health care providers is associated with failure to follow medical advice [30]. This finding emphasizes the importance of being aware of the influence of values on seeking care. Individuals’ help-seeking behavior can be affected by fears of judgment and discrimination.

The participants in this study reported many difficulties associated with adjustment to life in Canada. They expected to find a fit between what they had before in their country of origin and what they had now in their host country, and were dissatisfied to find that this was not the case. This finding is not surprising and has been reported by others; acculturation of different ethnic groups occurs at different rates that are influenced by social class, background, and the individual’s abilities [31,32]. Goldlust and Richmonds proposed a model of the immigrant adjustment process [33]. They believed that many factors, such as individual factors (age and gender), pre-migration experience (reason for immigration), and post-migration factors (immigration status and unemployment) influenced immigrants’ adjustment to their host country. Health care providers caring for new immigrants need to ensure that their health assessments include appraisal of these factors so that appropriate services can be coordinated and that trust in the health care provider and the Canadian health care system is not compromised.

In this study participants who were educated and younger at the time of immigration, had previous experience of being in a country other than Iran, and knew English seemed to become integrated into Canadian society, became self-sufficient, and learned to use the health care system more quickly than others. In contrast, those who had gone through traumatic events such as imprisonment, torture, or living in refugee camps for a long time had difficulty in becoming integrated and self-sufficient. We also found that self-sufficiency and integration were reciprocal. Going through the process of accessing health care services not only helped immigrants meet their health needs but also helped them become integrated into life in Canada. Such positive experiences affected their physical and mental health. They gained mastery in how to deal with health issues in particular and with all aspects of life in Canada in general. They shared their experiences with others and so helped others go through these stages more quickly. As they learned about barriers in the health care system, their ability to navigate these barriers improved. This raises the question of whether ability to navigate the health system successfully can be viewed as an indicator of integration.

The results of this study show that Iranian immigrants who were self-sufficient and able to navigate successfully the process of accessing health services did not give up their culture but instead move back and forth between their own and the Canadian culture in a manner consistent with biculturalism and acculturation. Studies of Iranian immigrants in Canada showed that some factors such as educational background, age at entry into the host country, length of residence, gender, and level of self-esteem are positively associated with the level of acculturation [4,34]. As Freire has pointed out, becoming integrated is an ongoing process, and it helps one acquire power over one’s life world and thus become liberated [35]. Having power is important because it helps people to understand, interpret, and shape their lives. Integration and adjustment to new lifestyles, access to health care services, and the ability to maintain the use of health care services are intertwined. Geiger has stated that because more than ever we are dealing with diverse populations, providing health care that is free from bias is a vital responsibility for health care providers [36]. Cultural diversity is one of the dominant attributes of Canada, and the ability of the Canadian health care system to respond to such diversity significantly influences the quality of care. As Glouberman stated, “If you look at people’s unease about healthcare system, it’s not because they have found it less than satisfying to use. It has to do with their fear that it won’t be there if they need it” [37].

The findings of this study highlighted immigrants’ needs for systems that are culturally and linguistically competent but that also recognize the importance of the immigrant’s ability to acquire language skill and cultural knowledge of mainstream society. Health care and social services should be tailored to facilitate this process. This means helping immigrants make decisions based on the personal significance of their historical, cultural, and social world, as individuals construct and reconstruct their reality. Each person's reality is unique, and everyone is the author of his or her own life. Individuals make health-related choices from within their own reality. Therefore, it is vital that immigrants’ viewpoints be taken into account. It is also important, however, that social services that enhance language acquisition and cultural knowledge of the host society are available to newcomers. Integration implies a reciprocal process that is both an immigrant and a host society responsibility.

The Iranian Immigrant Access Model to Canadian Health Care Services (IIAMCHCS) Figure 1 is congruent with the IOM model in most respects. It does however give greater centrality to what the IOM model proposes as mediating factors (appropriateness, efficacy of treatment, quality of providers and patient adherence) [38]. While such factors do not directly affect the initiation of first contact with health care services, our research suggests that without appropriate, effective, acceptable and responsive services, patients may decide not to follow recommendations, return for follow-up, or access such services in the future. In this new model, the definition of access is expanded to include such factors. The complexity of what is actually happening is revealed in our data. Studies on health services utilization show that new immigrants are “underusers” of the health care system because of societal or cultural barriers, or because the existing services do not meet their needs [39]. This study revealed, however, that immigrants who were struggling to access health care services sometimes misused, overused, or failed to use the provided services. In addition, the IOM model of access is a one-way model, whereas the model proposed in this study has many feedback loops. The process reported in this study was not linear. This study showed that the ability to access health care services became a vehicle for promoting integration. The better integrated the individuals were, the better their access to health care services was.

In addition, with respect to the notion of the global village and globalization, the world is changing. According to Freedman, “One of the implications of globalization is that virtually no culture is untouched by others.” ( [40] p.437). It is imperative for health care providers to understand that culture is socially constructed and changes over time. It is impossible to learn about all cultures, as they are constantly changing, but there is a simple, practical solution to this. As citizens of a global village, we have to be aware of this phenomenon and we have to be open to others’ preferences and expectations rather than making assumptions based on what is usually an inadequate understanding of the manifestations of culture in specific clients. Although transnationalism is considered a product of globalization and helps immigrants to find their way temporarily [41], because it keeps them close to their ties and far from interacting with mainstream host countries, it might have a negative effect on immigrants’ integration in host countries and their access to health care services. As opposed to transnationalism, which keeps immigrants close to their homeland, a culturally competent health care system facilitates and encourages learning about cultures, understanding similarities and differences among cultures, and sharing of cultures by both immigrants and their native-born counterparts. In providing culturally competent care, health care providers need to be aware of cultural expectations and should know how to engage in discussions to clarify individual patient priorities.

In nursing programs, our mission is to help students apply their knowledge in a practical realm, at the community level, and as research partners, educators, and policy makers at institutional and organizational levels. Given the cultural diversity of Canada, it is important to have curriculum context about culture and its effects on health in nursing programs. Nursing programs should seek opportunities for students to work with immigrants, particularly those who have not yet acquired language skills.

Students and faculty members should have the opportunity to examine their ideas, opinions, and even their prejudices, both through introductions to different cultures in their classrooms and through diversity in their colleagues and peers. By doing this, before working as graduate nurses, they gain awareness of the issues and can share strategies that they applied to solve or overcome conflicts in their work with their clients or other health care providers. In addition, within the health care system, matching client and health care provider by language and ethnicity helps immigrants to trust, seek care, and follow their treatment. In this study, participants appreciated the idea of having an Iranian health care provider. They had no preference concerning gender, as in Iran having a male or a female physician is a matter of choice. Therefore, hiring providers who are immigrants from different countries can help linguistically-culturally diverse populations trust the health care services.

The findings of this study suggest that providing information regarding resources and services offers immigrants the opportunity to make their own decisions concerning health care services and also to take an active role in their treatment. One of the reliable resources that participants in this study appreciated the most was the public library, along with assistance from librarians, a strategy and resource that has not been mentioned in previous studies. It would be interesting to explore if this is a common strategy for immigrants from other groups. If so, libraries and librarians could get enhanced holdings and training regarding the Canadian health care system and how to access it. A useful resource could become more attuned to specific access needs of newcomers.

Last but not least, even if health care services are committed to helping immigrants develop language skills, there will still be some occasions when an interpreter is required. In this case, medical interpreters and cultural brokers who are trained for the job, know medical terminology, and have good knowledge of the languages and cultures of both the patients and the dominant society should be employed. Although interpreters/cultural brokers and patients might be members of the same community, because they are hired by a designated organization, confidentiality is promised and patients are more likely trust to them. This would build trust and improve encounters between patients and health care providers. Because professional interpreters/cultural brokers are familiar with the cultures and languages of both patients and the dominant society, they exchange and translate as accurately as possible, which is beneficial to both patients and physicians. This affects health care services directly and, over time, is beneficial to the whole of society through the creation of a healthier population. Although the cost for the health care system to train medical interpreters and cultural brokers and create jobs for them would be high, it may be less than the cost of misdiagnosis or of not receiving treatment until a medical crisis happens, particularly for linguistic groups commonly seem in a specific health care setting. Research directed to cost-benefit analysis of such services is warranted.

It is suggested that researchers need to consider gender, host-language proficiency, familiarity with research processes, and comfort with the study in selecting research liaisons [42]. Considering the importance of gender as a social determinant of health, future studies need to examine the effect of gender on immigrants access to health care services in Canada. Focusing on migration and ethnic disparities, research scholars believe that it is also important to examine the reason and the cause of “why some migrant groups experience poor outcomes and why others do not” ( [43] p.248). Further study is needed to examine the influence of gender in the process of accessing health care services and integration. In studying immigrants and refugees, some scholars believe that researchers need to go beyond sex, gender or sexual orientation and ethnicity. Vissandjee and her colleagues suggest that researchers need to use intersectionality methodology in embracing sex, gender, ethnicity and migration as social determinants of health in order to inform health policy makers, improve strategies and prevent persistent disparities in health [44].

In conclusion, this study’s findings reveal intertwined and complex phenomena related to accessing health care services. We found that many elements, including language proficiency, cultural differences, education, previous experiences, financial status, age, knowledge of the host country’s health care services, and insider and outsider resources, influenced our participants’ ability to access health care services effectively and appropriately. Although this study showed that the issues concerning access to health care services faced by Iranian immigrants are similar to those experienced by others, comparative research involving immigrants from different countries is needed to ascertain similarities and differences. It is hoped that this study will offer some direction for all health care providers and policy makers in their efforts to provide accessible, appropriate, effective, responsive, and acceptable care to immigrants.

### Limitations

Because participants were recruited from a population of Iranian immigrants in a mid-sized city with a smaller Iranian population than in Toronto, Vancouver, and Montreal, one limitation of this study is related to the representativeness of the sample. Iranian immigrant populations are not homogenous; in larger populations, the results might be different. The second limitation is the level of education. Almost all of the participants were well educated, and so the results might have been different if people with lower levels of education had participated in this study. Finally, this study involved only Iranians who could speak and were fluent in Farsi (Persian). Iranians who spoke Balouchi, Kurdish, Arabic, Lori, Azari, and Gilaki as their mother tongue and were not fluent in Farsi or English were excluded from this study.

## Competing interests

The authors declare that they have no competing interests.

## Authors’ contributions

MD was fully responsible for conducting the study and writing the manuscript. KO approved the method of the study and LO reviewed and provided some revisions to the manuscript, particularly in relation to immigrant health context. All authors have read and approved the final manuscript.

## Supplementary Material

Additional file 1 **Appendix A:** Demographic Data. **Appendix B:** Initial Interview Questions. **Appendix C:** Example of Coding In English and Farsi. Click here for file
