# Supplementary material for: A study of Iranian immigrants’ experiences of accessing Canadian health care services: a grounded theory
Source: Int J Equity Health. 2012 Sep 29;11:55. doi: 10.1186/1475-9276-11-55 (PMC3519565; doi:10.1186/1475-9276-11-55)
Supplement: Additional file 1 — Appendix A: Demographic Data. Appendix B: Initial Interview Questions. Appendix C: Example of Coding In English and Farsi. [file 1475-9276-11-55-S1.doc]

Appendix A: Demographic Data

| AGE | GENDER | ARRIVAL IMMIGRATION STATUS | YEARS IN CANADA | EDUCATION |
| --- | --- | --- | --- | --- |
| 34 | M | Immigrant | 7 Years | Bachelor |
| 30 | M | Immigrant | 5 Years | Master |
| 25 | M | Refugee | 3 Years | Bachelor |
| 39 | M | Unknown | 13 Years | Master |
| 42 | M | Unknown | 10 Years | Master |
| 27 | M | Immigrant | 3 Years | Bachelor |
| 49 | F | Immigrant | 14 Years | High School |
| 39 | F | Immigrant | 7 Years | High School |
| 45 | F | Immigrant | 15 Years | Doctorate |
| 40 | F | Refugee | 12 Years | Master |
| 27 | F | Unknown | 4 Years | High School |
| 28 | F | Unknown | 3 Years | High School |
| 30 | F | Refugee | 3 Years | Bachelor |
| 44 | F | Immigrant | 8 Years | Bachelor |
| 38 | F | Unknown | 4 Years | Master |
| 25 | F | Immigrant | 2 Years | Bachelor |
| 32 | F | Refugee | 7 Years | Master |

**Appendix B: Initial Interview Questions**

Thinking back to the first time you wanted to access health care services in Canada.

1. Tell me about your experiences after coming to Canada. How was that time like for you?
2. Tell me about a time you experienced a health problem as an immigrant. What was that time like for you? How did you find it?
3. Tell me, how did you overcome with it?
4. As you think about your experiences with Canadian health care services, tell me what you have experienced as an immigrant?
5. How are things for you now?
6. What has been the most challenging experience about accessing Canadian health care services as an immigrant?
7. Can you tell me what you have learned from this experience?
8. Is there anything else about your access to Canadian health care services you would like to share with me?

Appendix C: Example of Coding In English and Farsi

| Coding in English | Interview in English | Interview in Farsi | Coding in Farsi |
| --- | --- | --- | --- |
| knowledge of Canadian health care services  Losing self -confidence  Feeling Helpless | …I didn’t know what to do…I had lost my self-confidence…I felt helpless. | .. نمیدونستم چیکار کنم.. اعتماد به نفسم رو از دست داده بودم... احسا س بدبختی و بیچارگی میکردم | احساس سر درگمی کردن احساس ناتوانی و درماندگی (بدبختی و بیچارگی) کردن  نا آگاهی از سیستم بهداشتی درمانی در کانادا |
